# Predicting seasonal influenza outbreaks with regime shift-informed dynamics for improved public health preparedness

**DOI:** 10.1038/s41598-024-63573-z

**Published:** 2024-06-03

**Authors:** Minhye Kim, Yongkuk Kim, Kyeongah Nah

**Affiliations:** 1 Department of Mathematics, Kyungpook National University, Daegu, 41566 Republic of Korea; 2 Busan Center for Medical Mathematics, National Institute for Mathematical Sciences, Busan, 49241 Republic of Korea

**Keywords:** Infectious diseases, Applied mathematics

## Abstract

In this study, we propose a novel approach that integrates regime-shift detection with a mechanistic model to forecast the peak times of seasonal influenza. The key benefit of this approach is its ability to detect regime shifts from non-epidemic to epidemic states, which is particularly beneficial with the year-round presence of non-zero Influenza-Like Illness (ILI) data. This integration allows for the incorporation of external factors that trigger the onset of the influenza season-factors that mechanistic models alone might not adequately capture. Applied to ILI data collected in Korea from 2005 to 2020, our method demonstrated stable peak time predictions for seasonal influenza outbreaks, particularly in years characterized by unusual onset times or epidemic magnitudes.

## Introduction

Influenza is mostly prevalent in the winter months, from May through October in the southern hemisphere and October through May in the northern hemisphere, and in the rainy season in tropical and subtropical regions, or year-round with no distinct season^1–3^. Outbreak characteristics, such as peak time and intensity, also vary considerably year-to-year so that such differences make timely healthcare preparedness measures, including resource allocations, hospital staffing, and dissemination of alerts, challenging^4^. The ability to predict the influenza peak several weeks in advance can be helpful for timely preventive public health planning and responses to mitigate these outbreaks^5,6^, and flu forecasting can significantly reduce the disease burden on the population^7–10^.

Several of the major approaches applied to modeling influenza transmission and dynamics have been applied to the forecasting of influenza outbreaks^11^. In 2013, the US Centers for Disease Control and Prevention (CDC) initiated a yearly nationwide influenza forecasting challenge^6,12^. The CDC has set predictive targets for public health decision-makers in the United States regarding the influenza season through real-time forecasting (1–4 weeks ahead). One particular emphasis of this consortium was not just simple all-or-nothing forecasts, but rather a probabilistic forecasting framework (i.e., estimating the likelihood of the influenza season reaching its peak within the next week or 2, etc.). The predictive outcomes encompass two types: temporal outcomes (e.g., peak time, duration of the epidemic) and intensity outcomes (e.g., peak magnitude, attack rates)^13,14^. A number of algorithms are developed in an attempt to forecast flu outbreaks^15–20^. Besides statistical approaches, many of those include compartmental models in their algorithms. In some studies, influenza is predicted using compartmental model with filtering method, which is a mathematical and statistical method that combines observation (data) and prior by the compartmental model to generate a posterior (new estimate)^18,21,22^.

Influenza forecasting requires predictive models parameterized by influenza surveillance data^18,23,24^. Often we utilize ILI surveillance data^25^, which represents the number of patient visits related to influenza-like illness as a proportion of the total number of patient visits. ILI cases are observed year-round, posing challenges in pinpointing the onset of the season. Identifying season onset, from when cases begin to increase exponentially is crucial for estimating the epidemic’s growth rate. The season onset varies by region and from year to year, and it depends on external factors such as viral evolution or environmental and social factors^26^. Several algorithms are designed to trigger official alerts of season onset. In a flu alarm system, the threshold is typically derived from historical ILI data and represents a level above the baseline where increased disease activity is expected. For example, the regional onset time of an epidemic is defined as the first of two consecutive weeks during which the incidence rate of ILI surpasses 150 cases per 100,000 inhabitants^27^. Alternatively, the onset of wintertime influenza is defined as the day when the observed excess P &I mortality rate, due to respiratory and influenza-related causes, exceeds the prescribed threshold level for the previous two weeks. For instance, setting the threshold at 0.01 deaths per 100, 000 people per day, the day when the mortality rate exceeds this threshold is considered the start of the influenza season^28^. More sophisticated methods, such as Bayesian Markov Switching Models-a type of regime shift model-have been suggested to detect epidemic onset^29,30^. This method divides a series of incidence data into epidemic and non-epidemic stages and then identifies the epidemic onset.

When employing mechanistic models for flu forecasting, the estimation of model parameters is necessary. These parameters may exhibit year-to-year variations, particularly transmission rate may fluctuate based on factors such as the circulating flu strain in the season. For parameter estimation, real-time surveillance data reflecting the flu incidence is utilized. Determining the appropriate time window for training the model requires knowledge of the season onset, which currently lacks a standard method of determination. In most influenza predictions, the season onset is either fixed or determined based on an incidence threshold: Nevertheless, to our knowledge, this approach has not been utilized for peak prediction.

In this study, we employ the existing Bayesian Markov Switching approach to identify the onset of influenza outbreaks from 2005 to 2020 in Korea. Using estimated yearly season onset and ILI surveillance data, we then predict influenza peaks by fitting the SIR model to a few weeks of data since the onset weeks we identified. The accuracy of our suggested approach in predicting peak time is compared with those obtained using different assumptions for the season onset.

## Material and methods

### Data

**Figure 1:**
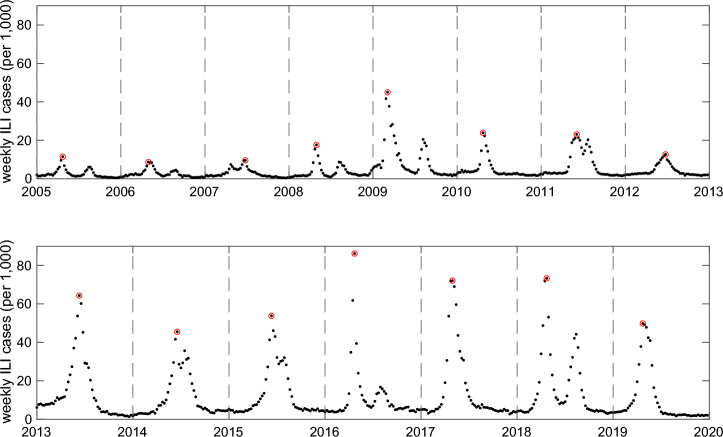
Weekly ILI cases per 1000 outpatients in South Korea from week 36 of each year to week 35 of the subsequent year, covering the period from 2005 to 2020.

**Figure 2:**
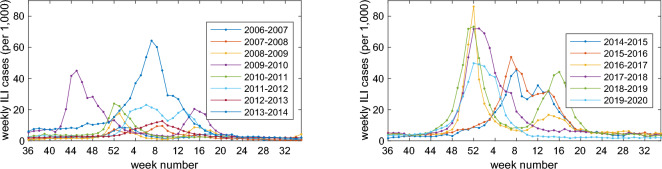
Weekly ILI cases per 1000 outpatients in South Korea from week 36 of each year to week 35 of the subsequent year, covering the period from 2005 to 2020.

For effective influenza forecasting, we utilize the data that can be collected in a timely manner. The Korea Disease Control and Prevention Agency (KDCA) operates an influenza monitoring system year-round, from September to August of the following year, in anticipation of the winter epidemic. It involves a network of outpatient clinics, which report the number of patients they have treated for influenza-like illnesses (ILI) on a weekly basis. The weekly ILI consultation rate in Korea, defined as the number of patient visits for influenza-like illness per 1000 outpatient visits, is depicted in Figs. 1 and 2. Though the temporal trends of ILI incidence from 2005 to 2020 reveal a consistent pattern of winter-to-spring spread, the timing for the season onset and the annual epidemic peak vary across years. To estimate the onset and peak time of the epidemic, we utilize the weekly ILI data available in the Korea Disease Control and Prevention Agency (KDCA) website^31,32^.

### Step 1: Detection of the season onset

As a preliminary step in our peak time prediction, we determine the onset of the seasonal outbreak in each year. For the comparative analysis of the accuracy of peak time prediction using different methods in the detection of the season onset, we present three different ways to detect season onset, namely Markov switching, threshold-based detection, and fixed-time detection.

- Fixed-time detection In detection approach, we follow the convention of using October 1 (week 40) as the onset time for each year.
- Threshold-based detection Although previous research has utilized threshold-based approaches to estimate the onset time of the flu season, there is no standard method for setting the threshold. In this study, the onset time is defined as the first week when the ILI rate exceeds a certain threshold value.
- Markov Switching In Ref.^30^, Hidden Markov models are utilized to distinguish between epidemic and non-epidemic phases. We adopt this approach to identify the season onset, which we define as the first week of regime shifts from non-epidemic to epidemic, starting from the beginning of the influenza season in a given year.

For $i \in \{1,\ldots ,52\}$ and $j \in \{1,\ldots ,15 \}$, we define $Y_{i,j}$ as the observed difference of the ILI rates between week $i+1$ and week *i* in year *j*, and $Z_{i,j}$ as an unobserved random variable that indicates the phase of the system: 1 for epidemic and 0 for non-epidemic. To infer the unobservable state $Z_{i,j}$ using the observed data $Y_{i,j}$, we consider a two-state Markov chain of order 1 with the transition probabilities defined as follows: $k,l \in \{0,1\}$, $P_{k,l}=P(Z_{i+1,j}=l|Z_{i,j}=k),$ where $P_{k,0}+P_{k,1}=1$. The conditional distribution of $Y_{i,j}$ is determined based on whether the system is in an epidemic or non-epidemic phase, noting that non-epidemic dynamics typically feature small random fluctuations around zero, while epidemic dynamics are characterized by larger and more correlated changes^30^. We model the conditional distribution of $Y_{i,j}$ as follows: for $i \in \{2,\ldots ,52\}$ and $j \in \{1,\ldots ,15 \}$,

$$ \begin{aligned}    & Y_{{1,j}} |(Z_{{1,j}}  = 0)\sim N(0,\sigma _{{0,j}}^{2} ), \\     & Y_{{1,j}} |(Z_{{1,j}}  = 1)\sim N(0,\sigma _{{1,j}}^{2} ), \\     & Y_{{i,j}} |(Z_{{i,j}}  = 0)\sim N(0,\sigma _{{0,j}}^{2} ), \\     & Y_{{i,j}} |(Z_{{i,j}}  = 1)\sim N(\rho Y_{{i - 1,j}} ,\sigma _{{1,j}}^{2} ), \\  \end{aligned}  $$

where *N* denotes a normal distribution. Note that we only need to estimate $\rho$, $P_{0,0}$, $P_{1,1}$ and $\{{\sigma ^2_{0,j}, \sigma ^2_{1,j}: j = 1,\cdots ,15}\}$. To estimate the parameters of the model, we employ the Bayesian method with prior distributions

$$ \rho \sim U( - 1,1),\quad P_{{0,0}} \sim B(0.5,0.5),\quad P_{{1,1}} \sim B(0.5,0.5). $$

here, *U* denotes a uniform distribution and *B* denotes a Beta distribution. Additionally, considering that $\sigma ^2_{0,j}$ should be lower than $\sigma ^2_{1,j}$ because it only reflects random variations rather than the effects of an epidemic, we express our prior knowledge about $\{{\sigma ^2_{0,j}, \sigma ^2_{1,j}: j = 1,\ldots , 15}\}$ via the following hierarchical structure:

$$ \sigma _{{0,j}} \sim U(\theta _{{low}} ,\theta _{{mid1}} ),\quad \sigma _{{1,j}} \sim U(\theta _{{mid2}} ,\theta _{{\sup }} ), $$

where $\theta _{low} \sim U(a,b)$, $\theta _{mid1} \sim U(\theta _{low},b)$, $\theta _{mid2} \sim U(\theta _{mid1},b)$, $\theta _{sup} \sim U(\theta _{mid2},b)$ with given numbers $a=0.1$ and $b=20$.

JAGS (Just Another Gibbs Sampler) is used for the parameter estimation and inference on the hidden states $Z_{i,j}$. Note that we only used parts of ILI data (2005–2013) for the parameter estimation leaving the later parts (2014–2020) for the purpose of testing. Estimated parameters of the Markov Switching model are presented in Supplementary Table S1.

Finally, the posterior probability of a given week being in the epidemic phase is determined by taking the average of the samples of epidemic states $Z_{i,j}$ of that week. If the posterior probability is above 0.5, then the week is classified as the week in the epidemic phase. Otherwise, it is classified as non-epidemic phase. We define the season onset to be the first week that is identified to be in the epidemic phase starting from week 36 of that year. See Supplementary Fig. S1 for the comparison of ILI epidemic curve and the posterior probabilities of the epidemic phase.

### Step 2: Prediction of outbreaks peaks

To estimate the peak of a given influenza season, we feed weekly ILI data into SIR model, starting from the estimated week of season onset. The rationale behind this approach is that epidemic curves tend to exhibit exponential growth in the initial phase, and previously estimated season onset provides an estimate of when the exponential growth phase begins. By comparing the ILI curve starting from the season onset with the infection curve of the SIR model, we can parameterize the SIR model and obtain a model-simulated peak of the season.

The SIR model describes the dynamics of Susceptible (*S*), Infectious (*I*) and Recovered (*R*) population with the following equations:

$$ \frac{{dS}}{{dt}} =  - \beta S\frac{I}{N},\quad \frac{{dI}}{{dt}} = \beta S\frac{I}{N} - \sigma I,\quad \frac{{dR}}{{dt}} = \sigma I, $$

where $N = S(t)+I(t)+R(t)$. We employ fixed values for the recovery rate $\sigma =1/7$, based on the assumption that the typical duration of influenza illnesses is around 1 week^19^. Given that the ILI data represents the number of patients with influenza-like symptoms per 1000 outpatients, we let $N=1000$ to ensure a direct comparison between the observed ILI data and the model simulation. To obtain estimates for the transmission rate $\beta$ and the initial values $I(t_0)$ and $R(t_0)$ for a given year with an estimated time of season onset $t_0$, the modeled incidence of the SIR model is fitted to 3–6 weeks of weekly ILI data starting from the onset week. This is done separately for each year with a distinct time of onset ($t_0$), using the least square method to obtain distinct values for $\beta$, $I(t_0)$, and $R(t_0)$ in each year. The initial number of susceptible individuals is calculated as $S(t_0)=N-I(t_0)-R(t_0)$. Using these estimated initial values and parameters, we then obtain the model-derived peak of the season through the numerical solution of *I*(*t*).

## Results

**Figure 3:**
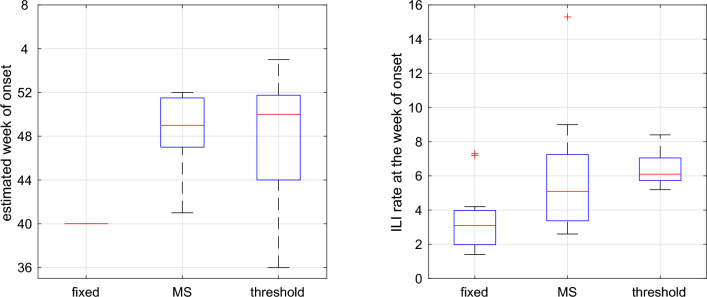
Boxplot for the season onset (left panel) and the ILI incidences at the season onset (right panel), using different distinct methods to estimate the season onset: fixed-time detection (fixed), Markov Switching (MS) and threshold-based detection (threshold).

Figure 3 presents a boxplot comparing the week of season onset and the ILI incidence at the detected onset using three methods: Markov switching, threshold-based detection, and fixed-time detection. A comparison of estimated season onset reveals that threshold-based detection yields the highest variance in season onset, while ILI at the estimated season onset exhibits low variance. On the other hand, the onsets detected with Markov Switching exhibit smaller variances in onsets but show higher variances in ILI at the onsets compared to the threshold-based detection method.

**Figure 4:**
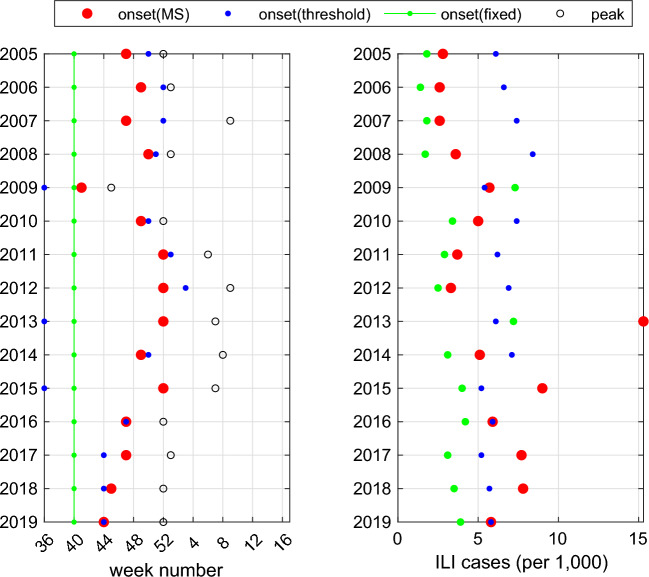
The left panel displays the yearly onset of seasonal outbreaks detected by Markov Switching (red dots), threshold-based detection (blue dots), and fixed-time detection (green dots). The y-axis indicates the beginning year of the influenza season. The black circles represent the peak times of the outbreaks in each season, defined as the maximum ILI value. For the threshold-based detection, 5.1 (per 1000) is used as the threshold ILI rate. The right panel shows the weekly ILI rate at the estimated season onset.

The left panel of Fig. 4 depicts the yearly onset of seasonal outbreaks estimated by three methods. Fixed-time detection yields the earliest estimated season onset in most of the years among the three methods. Onset weeks estimated by threshold-based detection exhibit higher variance compared to Markov Switching across the years due to substantial differences in epidemic sizes from year to year. Note that threshold-based detection sometimes detects the onset too close to the peak time, particularly in years with small epidemic sizes (e.g. seasons starting with years 2005 and 2006). This highlights the limitation of the threshold-based method in seasons with small epidemic sizes. Although the onset can be detected in earlier weeks with lower threshold values, this turned out to yield poor performance in peak time prediction, see Supplementary Fig. S2.

With the estimated season onset, we fit SIR model to 3–6 weeks of weekly ILI data starting from the onset week to obtain the model-derived outbreak peak. We then evaluate the accuracy of the peak time prediction by calculating the difference between the model-derived peak time and the peak time observed in the data, which is the highest ILI value reported during each season.

**Figure 5:**
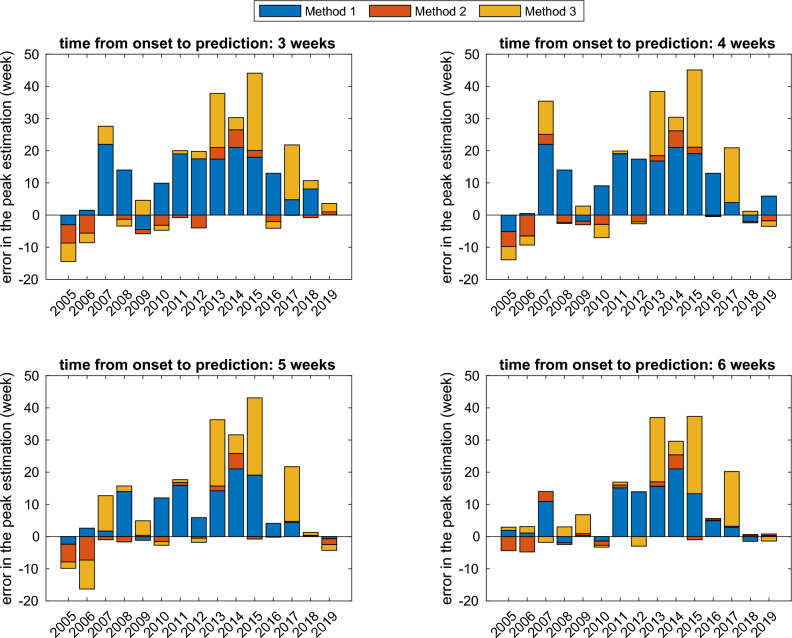
Errors of peak time prediction with respect to different methods used to detect season onsets and at different times of prediction. The x-axis represents the starting year of the flu season onset. On the y-axis, the prediction error is depicted, which is determined by subtracting the estimated peak from the peak of the ILI curve. Labels ‘Method 1’, ‘Method 2’, and ‘Method 3’ denote the peak time predictions based on fixed-time, Markov Switching, and threshold-based estimation of season onset, respectively.

**Figure 6:**
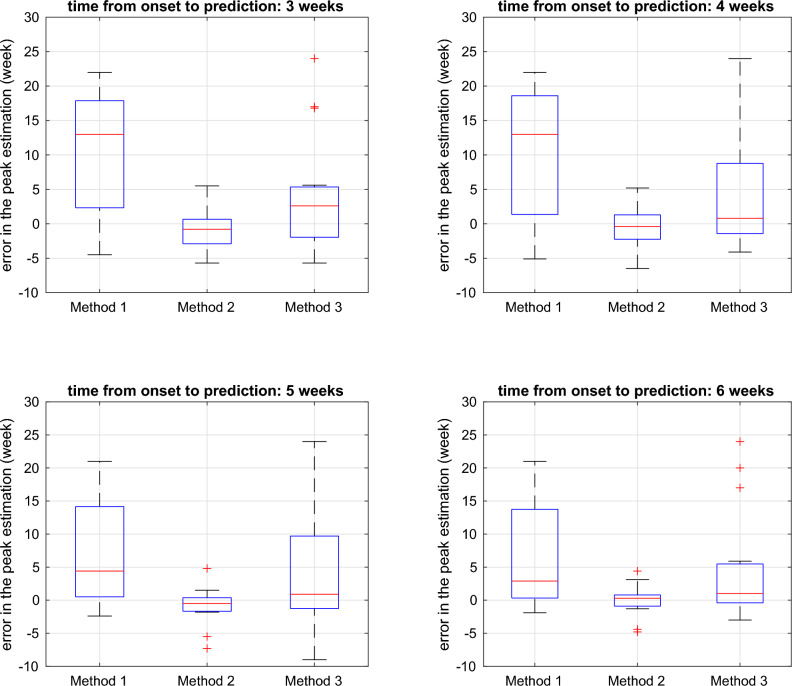
Boxplot for the error of peak time prediction with respect to different methods used to detect season onsets and at different times of prediction. Labels ‘Method 1’, ‘Method 2’, and ‘Method 3’ denote the peak time predictions based on fixed-time, Markov Switching, and threshold-based estimation of season onset, respectively.

Figures  5 and 6 illustrates the accuracy of peak time prediction using various methods for estimating the season’s onset. We have adopted the labels ‘Method 1’, ‘Method 2’, and ‘Method 3’ to denote the peak time predictions based on fixed-time, Markov Switching, and threshold-based estimation of season onset, respectively. Method 2, which utilizes the onset estimated by Markov Switching, demonstrated the highest accuracy and the least variance in error, surpassing the other two methods. Each panel in the figures presents the results for different prediction times, with later predictions showing higher accuracy. When the prediction was made 6 weeks after the season onset, method 2 predicted the peak time with less than 2 weeks of error for most of the flu season.

Moreover, we evaluated the performance of the threshold-based detection method using various threshold values ranging from 1 to 5 (per 1000), and the results are reported in Supplementary Fig. S2 in terms of the accuracy of peak time prediction. The optimal threshold value for the threshold-based detection method was found to be 5 ILI per 1000 population. Setting the threshold higher than 5 can result in delayed onset detection, leading the peak time predictions to be made after the actual peak.

From the ANOVA table presented in Table 1, one can see that the group means of the absolute error of peak time prediction under different estimation methods are significantly different. The significance of the difference is more pronounced (with a p-value of less than 0.005) when the prediction is made earlier (i.e., 3–4 weeks from the onset to prediction).

**Table 1:** ANOVA table for the absolute error of peak time prediction with different estimation methods for season onset.

	Sums of squares	Mean square	F-value	p-value
Time from onset to prediction: 3 weeks				
Methods	628.9	314.45	8.61	0.0007
Residuals	1533.05	36.50		
Time from onset to prediction: 4 weeks				
Methods	625.05	312.52	7.6	0.0015
Residuals	1727.3	41.13		
Time from onset to prediction: 5 weeks				
Methods	309.17	154.59	3.87	0.0286
Residuals	1676.56	39.92		
Time from onset to prediction: 6 weeks				
Methods	243.72	121.86	3.21	0.0506
Residuals	1596.87	38.02		

**Figure 7:**
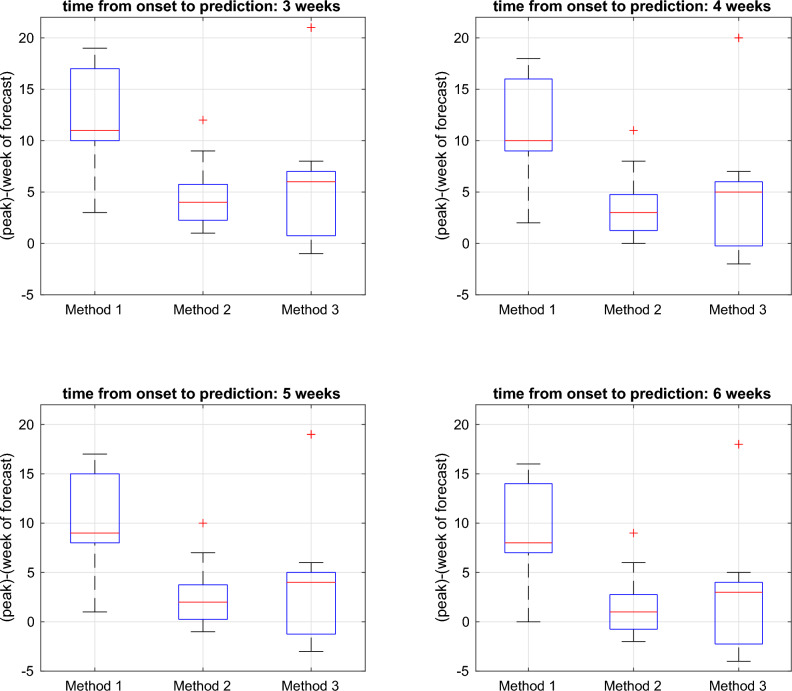
Boxplot for the error of peak time prediction with respect to different methods used to detect season onsets and at different times of prediction. Labels ‘Method 1’, ‘Method 2’, and ‘Method 3’ denote the peak time predictions based on fixed-time, Markov Switching, and threshold-based estimation of season onset, respectively.

Plots in Fig. 7 show the remaining time from the week of peak time prediction to the actual peak. A positive value indicates that the prediction was made before the peak time, while a negative value indicates that the prediction was made after the peak time. The fixed method (Method 1) naturally provides the earliest prediction. The threshold-based method (Method 3) has been found to yield a higher median compared to the Markov Switching-based method (Method 2), However, this method can lead to delayed predictions that occur after the peak has passed. On the other hand, the Method 2 exhibits the lowest variance in the remaining times from the week of prediction to the peaks, providing higher stability in the peak prediction. Specifically, when prediction is made 3 or 4 weeks after detected onset, it allows 2–5 weeks from the time of prediction to peak. When prediction is made 5 weeks after onset detection, there is a 75% chance that the prediction will be made before the peak, and when predictions is made 5 weeks after onset, more than 25% of the predictions are made after the peak.

## Discussion

Accurate and timely prediction of seasonal influenza peaks can help inform decisions about vaccination campaigns and other measures to control the spread of seasonal influenza. In this study, we propose a novel approach that combines Bayesian Markov Switching and a mechanistic SIR transmission model to predict seasonal peaks using ILI incidence data. The underlying concept is to leverage Markov Switching, which identifies the transition from a non-epidemic phase to an epidemic phase, to detect the onset of the epidemic curve where exponential growth begins. The key benefit of the Markov Switching in our context lies in its ability to detect regime shifts from non-epidemic to epidemic states, which is particularly challenging with non-zero ILI data throughout the year. By this integration of Markov Switching with a disease dynamics, we allow the model to catch external drivers of influenza season onset which purely mechanistic models may overlook. We use this identification to determine the starting point for the SIR model simulations.

By assessing the accuracy of various methods for estimating the peak time of seasonal influenza outbreaks, we find that peak time prediction utilizing the Markov Switching method outperforms the fixed-time method and the threshold-based method. The alternative methods exhibit limitations in providing stable predictions, particularly in cases where the onset occurs at an unusual time or the epidemic size deviates significantly. Specifically, when the prediction is made 3 or 4 weeks after the detected onset, the Markov Switching method typically allows for a 2–5 week period from the time of prediction to the peak. These findings align with previous studies that have demonstrated stable peak time forecasts 2–5 weeks before the actual peak^18,33^.

As a limitation of our study, we did not provide uncertainty intervals for the predicted peaks, which are essential for effective communication with the public and public health officials. To address this limitation, alternative methods beyond least squares should be considered to estimate parameters for disease dynamics and provide uncertainty estimates for the predicted peaks. It’s important to note that we do not claim Markov Switching as the superior tool for season onset estimation or peak prediction. Instead, we demonstrate the utility of the regime shift approach in informing the initial time of disease dynamics, offering stable predictions for epidemic peaks. This was illustrated using ILI data from Korea and compared with other methods, including threshold-based and fixed-time approaches. It remains an avenue for future research to evaluate the applicability of our approach with ILI data from different regions. Additionally, the potential use of other methods for season onset detection, such as the moving epidemic method (MEM)^34,35^, as input for disease dynamics remains to be investigated. Nevertheless, we conclude that incorporating our proposed approach into existing ensemble models for peak time prediction has the potential to enhance prediction accuracy.

### Supplementary Information

Supplementary Information.

## Data Availability

The datasets analysed during the current study are available in the Korea Disease Control and Prevention Agency (KDCA) website^31^.
