# Supplementary material for: Predicting seasonal influenza outbreaks with regime shift-informed dynamics for improved public health preparedness
Source: Sci Rep. 2024 Jun 3;14:12698. doi: 10.1038/s41598-024-63573-z (PMC11148101; doi:10.1038/s41598-024-63573-z)
Supplement: Supplementary file 1 — Supplementary Information. [file 41598_2024_63573_MOESM1_ESM.pdf]

Supplementary Information

Parameters and states of Markov Switching model

Parameter	Posterior mean	95% credible interval
θ_{low}	0.164	[0.11, 0.23]
θ_{mid1}	0.634	[0.509, 0.784]
θ_{mid2}	0.805	[0.614, 1.033]
θ_{sup}	6.541	[5.27, 8.38]
ρ	0.543	[0.441, 0.647]
$P_{0,0}$	0.938	[0.91, 0.962]
$P_{0,1}$	0.062	[0.038, 0.09]
$P_{1,0}$	0.085	[0.051, 0.125]
$P_{1,1}$	0.915	[0.875, 0.949]

TABLE S 1. Posterior mean and 95% credible interval for the parameters.

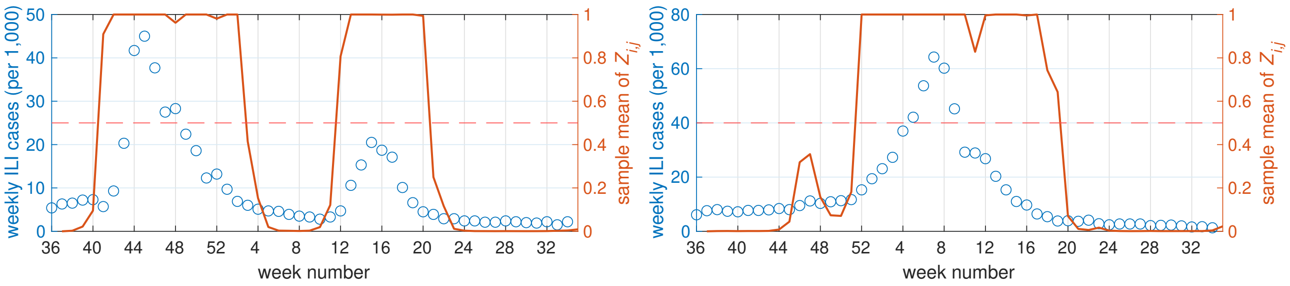

FIGURE S 1. Weekly ILI rates (blue circles) and the sample means of $Z_{i,j}$ (red line) which indicates the epidemic status of week i in year j. Left and right panel corresponds to 2009-2010 influenza season ($j = 5$) and 2013-2014 influenza season ($j = 9$), respectively.

Supplementary Table S1 displays the posterior means and 95% credible intervals of the model parameters which are determined by the ranges of parameters that account for 95% of the posterior distribution. Supplementary Fig. S1 present sample means of the hidden state $Z_{i,j}$ which indicates epidemic status of week i in year j.

Peak time prediction with threshold-based detection of season onset

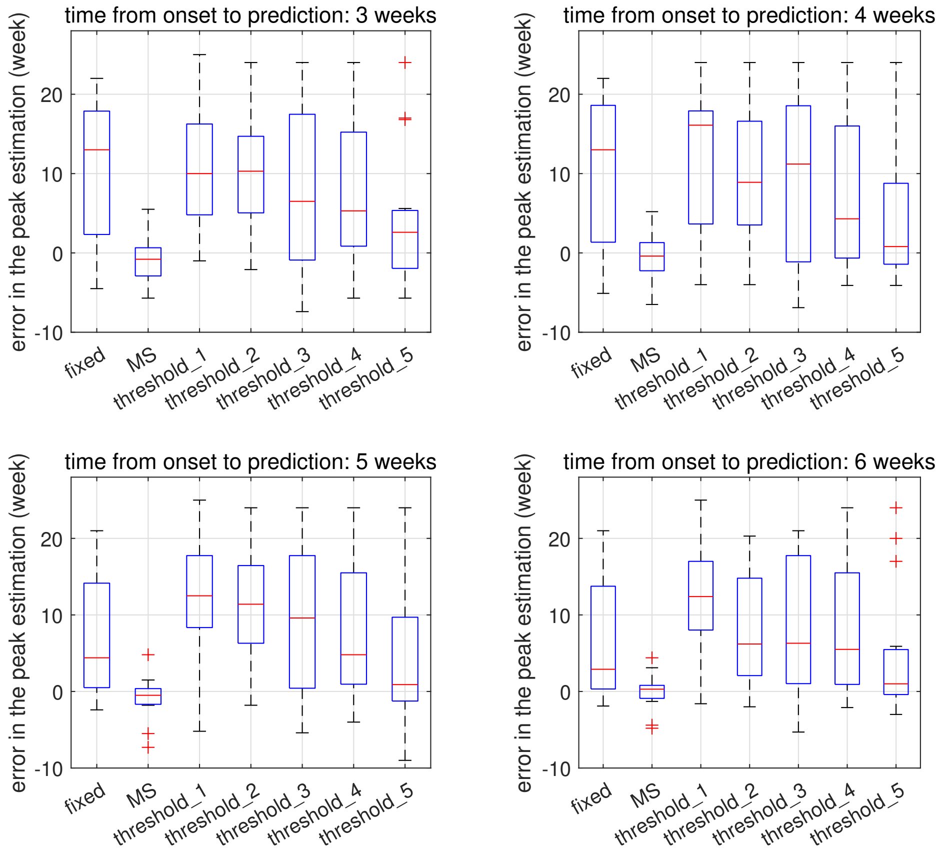

FIGURE S 2. Boxplot representing the error of peak time prediction across different methods of detecting season onsets, including the threshold-based detection method with different threshold levels.

The error of peak time prediction is presented in a boxplot that compares different methods of detecting season onsets, including the threshold-based detection method with threshold levels ranging from 1 to 5, as shown in Supplementary Fig. S2. It is generally observed that increasing the threshold for the threshold-based detection method from 1 to 5 results in better accuracy in predicting the peak time. However, it should be noted that setting the threshold too high can lead to negative errors. When the threshold is set too high, the season onset may be detected at later times, which leads to later predicted peaks than the actual peak.
